# Baseline Clinical Characteristics and Incidence of Chronic Thromboembolic Pulmonary Hypertension Patients in Latvia, 2019–2020

**DOI:** 10.3390/medicina59081426

**Published:** 2023-08-06

**Authors:** Ričards Kauliņš, Ainārs Rudzītis, Aivars Lejnieks, Dana Kigitoviča, Andris Skride

**Affiliations:** 1 Department of Internal Diseases, Riga Stradiņš University, LV-1079 Riga, Latvia; 2 Department of Rare Diseases, Pauls Stradiņš Clinical University Hospital, LV-1002 Riga, Latvia; 3 Department of Internal Diseases, Riga East Clinical University Hospital, LV-1079 Riga, Latvia

**Keywords:** chronic thromboembolic pulmonary hypertension, epidemiology, incidence, Latvia, national registry

## Abstract

*Background*: Chronic thromboembolic pulmonary hypertension (CTEPH) is a rare and progressive condition; however, the true characteristics of CTEPH are still unknown, as notable regional variations exist in terms of patients’ age, baseline hemodynamic data, and management choices. This report aims to investigate the baseline clinical characteristics, incidence, and risk factors associated with CTEPH patients in Latvia from 2019 to 2020. *Methods*: The data were analyzed from a prospective, nationwide, Latvian pulmonary hypertension registry for incident CTEPH cases. The patients’ clinical characteristics were assessed at the time of diagnosis. *Results*: During the course of this study, a cohort of 13 patients with CTEPH were included for analysis. Among the enrolled CTEPH patients, most exhibited low exercise and functional capacity, with a median (±IQR) 6 min walk distance of 300.0 (±150.0) m. The median values (±IQR) for mean pulmonary artery pressure and pulmonary vascular resistance were 40.0 ± 13.0 mmHg and 7.35 ± 2.82 Wood units, respectively. The most common risk factors for CTEPH were a history of acute pulmonary embolism and a blood group other than O. *Conclusions*: The findings of this report revealed the characteristics of the Latvian CTEPH population, indicating that a significant proportion of patients are elderly individuals with multiple comorbidities.

## 1. Introduction

Chronic thromboembolic pulmonary hypertension (CTEPH) is a rare and progressive condition which is hemodynamically characterized by pre-capillary pulmonary hypertension (PH) [1,2]. The European Society of Cardiology’s newest PH recommendations, released in 2022, describe pre-capillary PH as a mean pulmonary arterial pressure (mPAP) >20 mmHg, pulmonary capillary wedge pressure (PCWP) 15 mmHg, and pulmonary vascular resistance (PVR) >2 Wood units (WU) [2]. The present report is on CTEPH patients in 2019 and 2020 who were enrolled before the most recent PH update; thus, these CTEPH patients were diagnosed based on the 6th World Symposium on Pulmonary Hypertension Task Force, which defined CTEPH as a mPAP 20 mmHg, PCWP 15 mmHg, and PVR > 3 Wood units [3], using recommendations for the confirmation of chronic thromboembolic obstructions from the European Society of Cardiology (ESC) and the European Respiratory Society (ERS) PH guidelines in 2015 [4].

According to national and international CTEPH registries, the reported incidence of CTEPH is reported to be between 0.3 and 39 cases per million inhabitants [5,6,7]. Despite the fact that CTEPH is considered a rare disease, it still places a considerable burden on society due to excessive health care consumption, high-cost therapy, and higher mortality [8]. Over the last decade, significant progress has been made in the field of CTEPH. These breakthroughs include advancements in surgical, interventional, and pharmacological therapies, as well as extensive international and national registries [9]. These collective efforts have greatly enhanced our understanding of this disease’s epidemiology, management, and diagnostics [9]. However, the true characteristics of CTEPH in the current registries are affected by overlooked or unaccounted CTEPH patients in the general population of a given territory [1], as the majority of the registries fail to include all pulmonary hypertension centers of the territory in question. Furthermore, notable regional variations exist in terms of patients’ age, baseline hemodynamic data, and management choices concerning the three primary treatment modalities for pulmonary endarterectomy (PEA), balloon pulmonary angioplasty (BPA), and pulmonary arterial hypertension (PAH) therapy [1]. This demonstrates the importance of obtaining epidemiological data not only from large, worldwide registries but also from local national registries that consider the above-mentioned regional differences. This information could lead to a better worldwide knowledge of CTEPH. As a result, annual reports outlining the features of CTEPH patients might follow the changing dynamics of CTEPH cases over time, with the goal of identifying new trends and patterns.

The objective of this report was to present the baseline characteristics of incident CTEPH patients from the Latvian PH registry in 2019 and 2020, which includes all CTEPH cases recorded in Latvia.

## 2. Materials and Methods

The Latvian Pulmonary Hypertension registry is a single-center registry based at Pauls Stradiņš Clinical University Hospital (Riga, Latvia) and is the sole center dedicated to pulmonary hypertension in Latvia.

This report presents the baseline characteristics of newly diagnosed adult (age ≥ 18 years) CTEPH patients during the period from 1 January 2019 to 31 December 2020 in a Latvian PH center. Each patient who participated in the registry completed a written informed consent form for inclusion in the registry and the publication of the study data. Before signing the informed consent form, the nature of the study was explicitly described. This research was carried out in line with the 1975 Helsinki Declaration (revised in 2008). The research was approved by the Pauls Stradiņš Clinical University Hospital Ethical Committee (15 December 2009), ethical approval No. 151209-6L.

The CTEPH diagnosis was set accordingly in the presence of the following findings: mPAP > 20 mmHg, PAWP ≤ 15 mmHg measured via right heart catheterization (RHC) according to the 6th World Symposium on Pulmonary Hypertension Task Force [3], confirmation of chronic thromboembolic obstructions on computed tomography pulmonary angiography (CTPA), or classic pulmonary angiography as described in the European Guidelines [4] after at least three months of adequate anticoagulation therapy. The ventilation–perfusion (VQ) scan was not used for the confirmation of the diagnosis, as the procedure was not available in Latvia at the time of the patient enrollment. 

The data in this report were compiled at the PH center at the time of diagnosis and include demographics, vital parameters (blood pressure, heart rate, oxygen saturation (SpO_2_) in normal room air), comorbidities, CTEPH risk factors (mentioned in Table 2) concomitant medication, hemodynamical parameters obtained from RHC (mPAP, PVR, PCWP, right atrial pressure (RAP), cardiac index (CI) and cardiac output (CO)), World Heart Organization (WHO) functional class (FC), 6 min walk distance (6 MWD), brain natriuretic peptide (BNP), and CTPA data.

The baseline characteristics were reported using the median and interquartile range (IQR) as measures of central tendency and dispersion, respectively, given the small sample of CTEPH patients.

## 3. Results

Through the duration of this study, a total of 13 CTEPH patients were included in the Latvian PH registry. In total, 6 of the patients were diagnosed with CTEPH in 2019 and 7 were diagnosed in 2020. The CTEPH patient baseline clinical and hemodynamic characteristics are described in Table 1. The median (±IQR) BMI of the CTEPH patients was 24.0 (±7.0) kg/m^2^. A total of 3 patients had a BMI of 25–30 kg/m^2^, qualifying them as overweight, and 3 patients had a BMI greater than 30, qualifying them as obese at the time of enrollment. The 6 MWD was measured at the time of diagnosis to assess functional capacity. In total, 2 patients were not able to participate in the 6 MWD test due to their severely reduced exercise capacity. The median (±IQR) 6 MWD for the CTEPH patients who completed the 6 MWD was 300.0 (±150.0) meters, with a median distance (±IQR) of 285.0 (±52.5) meters in 2019 and 420.0 (±165.0) meters in 2020 (*n* = 5). The levels of brain natriuretic peptide (BNP) at the time of enrollment exhibited considerable variability, ranging from a minimum of 83.00 pg/mL to a maximum of 1881.84 pg/mL. Right heart catheterization was conducted to gather hemodynamic measures. Technical issues prevented us from taking hemodynamical measurements of PVR, CI, and CO in one of the patients. 

The assessment of risk factors for CTEPH revealed that the majority of the patients (10 out of 13) had a previous history of acute pulmonary embolism and (10 out of 13) possessed a blood group other than O. The assessed risk factors of the CTEPH patients are described in Table 2. 

A large proportion of the enrolled CTEPH patients had comorbidities associated with left heart disease, with the most frequent being coronary artery disease (8 out of 13 patients) followed by systemic arterial hypertension (6 out of 13 patients). The assessed concomitant diseases of the CTEPH patients are described in Table 3.

During the enrollment, 2 patients were deemed eligible for surgery and 11 patients were categorized as inoperable, of whom 3 patients received the BPA procedure. The main reasons for inoperability were primarily linked to the distal localization of the thromboembolic lesions (6 out of 11 patients), as well as factors like a poor clinical condition or a low benefit-to-risk ratio (5 out of 11 patients).

Due to the substantial waiting time for surgical intervention for CTEPH in Latvia, all the patients received off-label therapy using PAH treatments. In total, 12 patients were treated with monotherapy consisting of phosphodiesterase type 5 inhibitors (PDEi), while 1 patient received a combination therapy of PDEi and an endothelin receptor antagonist. Meanwhile, 2 patients later received a pulmonary endarterectomy, and 3 patients received the BPA procedure when it became available in Latvia in 2022. At the time of enrollment, the majority of the patients were predominantly using vitamin K antagonists (8 out of 13 patients) and new oral anticoagulants (NOACs) (5 out of 13 patients). The utilization of other therapies such as beta blockers, ivabradine, statins, etc., was indicative of the prevalence of comorbidities among the patients. The therapies received by the CTEPH patients at the time of enrolment are described in Table 3.

The population of Latvia was 1,919,968 inhabitants in 2019 and 1,907,675 inhabitants in 2020, among whom the number of adult inhabitants (≥18 years old) was 1,561,155 in 2019 and 1,548,218 in 2020 [10]. The pooled CTEPH incidence in Latvia in 2019 was 3.84 per million adult inhabitants (MAI) and 3.13 per million inhabitants (MI), while in 2020, it was 4.52 per MAI and 3.67 per MI.

## 4. Discussion

This report presents the baseline characteristics of CTEPH patients from the Latvian nationwide PH registry during the years 2019 and 2020. The incidence of CTEPH patients in Latvia during 2019 was 3.84 per million adult inhabitants (MAI) and 3.13 per million inhabitants (MI), whereas in 2020, it increased to 4.52 per MAI and 3.67 per MI. However, upon comparing these results with the previous reports, it is evident that the incidence of CTEPH in Latvia has been fluctuating in recent years, albeit with a slight overall trend towards decrease. The reported incidence for the Latvian CTEPH cohort was 5.1 per MI in time period of 2007–2016, 11.8 per MI and 14.5 per MAI in 2017, and 3.6 per MI and 4.5 per MAI in 2018 [11,12,13]. 

The confirmation of chronic thromboembolic obstructions was mainly performed using computed tomography pulmonary angiography (CTPA) or conventional pulmonary angiography. At the time of patient enrollment, the V/Q scan was not utilized in Latvia due to its unavailability. However, it is noteworthy that as of the beginning of 2023, the V/Q scan has become available in Latvia.

At the time of diagnosis, incident patients with CTEPH had a median (±IQR) mPAP of 40.0 (±13.0) mmHg and a high median (±IQR) PVR of 7.35 (±2.82) WU, which demonstrates that incident patients with CTEPH exhibit moderate to severe PH symptoms. Comparing these results with the previous reports of the Latvian CTEPH population, the mean (±SD) hemodynamic values for mPAP and PVR were 41 (±8) mmHg and 7.76 (±2.99) WU in 2017 and 38.0 (±11.7) mmHg and 8.0 (±6.5) WU in 2018, respectively [12,13]. This shows that the mean baseline hemodynamic values remain similar from year to year in the Latvian CTEPH population. Similar hemodynamic results were found when comparing these data to other European CTEPH registries: BNP-PL: mPAP 45.5 (±11.8) mmHg, PVR 7.7 (±4.6) WU; HOPE: mPAP 44.5 (±15.3) mmHg, PVR 8.0 (±5.0) WU; SPHAR: mPAP 46 (±17) mmHg; PVR 7 (±7) WU; and Germany: mPAP 43 (±10) mmHg, PVR 9 (±4.5) WU [14,15,16,17]. 

The results showed that most of the patients were classified as WHO FC II (3 out of 13 patients (21.1%)) and III (8 out of 13 patients (61.5%)), followed by FC IV (2 out of 13 patients (15.4%)), where the median (±IQR) 6 MWD was 300.0 (±150.0) m at the time of enrollment. However, these results indicate a significantly limited exercise capacity, comparing them with the data from the Latvian registry in 2018, i.e., the functional capacity of CTEPH patients has significantly improved. We can observe that 57.1% (four out of seven) of CTEPH patients in 2018 were classified as FC III and 42.9% (three out of seven patients) were classified as FC IV, with a mean (±SD) 6 MWD of 171.0 (±142.1) m [13]. Comparing the results of the current report with the data from other European CTEPH registries, Latvia has a higher proportion of patients in the FC IV category and noticeably lower 6 MWD results (BNP-PL: FC IV 6%; 6 MWD 334 (±166.4) m; HOPE: FC IV 1%; 6 MWD 347 (±220) m; SPHAR: FC IV 8%; 6 MWD 345 (±198) m) [14,15,16]. Although the results showing a significantly reduced exercise capacity would indicate severe disease progression at the time of enrollment, interestingly, comparing the results of the median (±IQR) RAP with the same registries, the Latvian CTEPH patients had a significantly lower median (±IQR) RAP 3.0 (±4.0 mmHg (mean (±SD) RAP: BNP-PL 7.7 (±4.6) mmHg; HOPE 8.0 (±5.0) mmHg; SPHAR: 7 (±7) mmHg) [14,15,16]. This indicates that the right ventricle function is preserved at the time of enrollment for CTEPH patients in Latvia; thus, the reduced functional capacity may be attributed to the patients’ underlying conditions rather than the disease’s progression. 

Among the CTEPH patients, the most common comorbidities observed included coronary artery disease (8 out of 13 patients (61.5%), systemic arterial hypertension (6 out of 13 patients (46.1%), dyslipidemia (6 out of 13 patients (46.1%), and thyroid disorders (5 out of 13 patients (38.5%). Comparing these results with those of other CTEPH registries, it was found that the prevalence of these comorbidities is higher in the Latvian CTEPH population [14,15]. The higher prevalence of these cardiovascular diseases in CTEPH patients could be attributed to the general Latvian adult population, which is burdened with a high prevalence of cardiovascular diseases. The annual report of Health Behavior among the Latvian Adult Population showed that in the age group of 55–74 years, which most of the enrolled CTEPH fell within (*n* = 9; 69.2%), the prevalence of systemic arterial hypertension was 45.5%, almost the same as the prevalence observed in the Latvian CTEPH population (46.1%) [18]. However, the prevalence of thyroid disorders in the same report in the age group of 55–74 years was 2.2%, which is noticeably lower than that observed in the Latvian CTEPH population (38.5%). Our results support the previous research that has linked thyroid disorders, particularly hypothyroidism and treatment involving levothyroxine, to CTEPH [19,20]. 

Almost all the CTEPH patients (11 out of 13) had a history of venous thromboembolism, where the most common risk factors for CTEPH were a history of acute pulmonary embolism (10 out of 13 patients) and a blood group other than O (10 out of 13 patients). Notably, other European studies have reported that approximately half of CTEPH patients have a history of deep vein thrombosis [14,15,21]. However, in our study, the incidence of a history of deep vein thrombosis was found to be significantly lower compared to the European registries. This disparity could be due to potentially undiagnosed deep vein thrombosis in the CTEPH patients.

All the CTEPH patients enrolled in this registry received off-label therapy using PAH treatments, where most of the patients received a monotherapy consisting of PDEi and one patient received a combination therapy of PDEi and an endothelin receptor antagonist. Although the approved treatment for inoperable and persistent/recurrent CTEPH patients is riociguat [22], this drug is currently not reimbursed in Latvia; thus, the patients had to receive off-label therapy using PAH treatments. At the time of enrollment, all the CTEPH patients were receiving anticoagulation therapy, with the majority prescribed vitamin K antagonists (8 out of 13), while the remaining patients were treated with NOACs (5 out of 13). The utilization of NOACs among the CTEPH patients was due to NOAC therapy’s reduced side effects and the convenience of NOAC dosing compared to vitamin K antagonists, especially in patients with a high prevalence of comorbidities. Despite the fact that there is no endorsement of NOACs in the pulmonary hypertension guidelines, recently, CTEPH centers and registries have reported an increase in the utilization of NOACs in CTEPH centers [23,24]. The utilization of other therapies such as beta-blockers, ivabradine, statins, etc., points to the burden of underlying conditions among CTEPH patients.

## 5. Conclusions

This report outlines the characteristics of the Latvian CTEPH population, revealing that the majority of Latvian CTEPH patients are elderly and burdened with multiple comorbidities. At the time of enrollment, substantial limitations in exercise capacity and functional ability were experienced by most of the CTEPH patients in Latvia. These observations emphasize the importance of comprehensive management strategies that target both the primary illness and its comorbidities in order to enhance the general quality of life of CTEPH patients in Latvia.

## Figures and Tables

**Table 1:** Baseline clinical and hemodynamic characteristics of Latvian CTEPH patients in 2019 and 2020.

Baseline Characteristics	All CTEPH Patients (*n* = 13), Median ± IQR	CTEPH in 2019 (*n* = 6), Median ± IQR	CTEPH in 2020 (*n* = 7), Median ± IQR
Age, years	67.0 ± 17.0	57.0 ± 18.5	68.0 ± 9.5
Female/male ratio	2.25	2	2.5
BMI, kg/m^2^	24.0 ± 7.0	25.5 ± 9.2	23.6 ± 6.2
BNP, pg/ml	582.00 ± 525.45	449.50 ± 464.69	586.65 ± 631.57
SpO_2_, %	97.0 ± 1.0	96.5 ± 1.0	97.0 ± 5.0
Functional status			
WHO FC n,			
I	0	0	0
II	3	1	2
III	8	5	3
IV	2	0	2
6 MWD, m	300.0 ± 150.0 (*n* = 11)	285.0 ± 52.5	420.0 ± 165.0 (*n* = 5)
Right heart catheterization			
mPAP, mmHg	40.0 ± 13.0	41.5 ± 10.5	39.0 ± 14.5
RAP, mmHg	3.0 ± 4.0	3.0 ± 3.5	3.0 ± 3.0
PVR, WU	7.35 ± 2.82 (*n* = 12)	8.67 ± 5.27 (*n* = 5)	6.80 ± 2.81
PCWP, mmHg	10.0 ± 8.5 (*n* = 12)	10.0 ± 0.0 (*n* = 5)	15.0 ± 9.0
CI, l/min/m2 ± SD	2.06 ± 0.70 (*n* = 12)	1.86 ± 0.54 (*n* = 5)	2.28 ± 0.67
CO, l/min ± SD	3.73 ± 2.06 (*n* = 12)	3.20 ± 2.04 (*n* = 5)	3.87 ± 2.04

Abbreviations: CTEPH—chronic thromboembolic pulmonary hypertension, IQR—interquartile range; BMI—body mass index; BNP—brain natriuretic peptide; SpO_2_—oxygen saturation; *n*—number of patients; WHO FC—World Health Organization Functional Class; 6 MWD—6 min walking distance; PVR—pulmonary vascular resistance; WU—Wood units; mPAP—mean pulmonary artery pressure; RAP—right atrial pressure; PVR—pulmonary vascular resistance; PCWP—pulmonary artery wedge pressure; CI—cardiac index; CO—cardiac output.

**Table 2:** Risk factors for CTEPH.

CTEPH Risk Factor	All CTEPH Patients, *n*	CTEPH Patients in 2019, *n*	CTEPH Patients in 2020, *n*
History of venous thromboembolism	11	6	5
History of acute pulmonary embolism	10	5	5
History of deep vein thrombosis	3	2	1
Recurrence of pulmonary embolism	3	2	1
Chronic inflammatory disease	0	0	0
Atrial septal defect	0	0	0
VA shunt	0	0	0
History of splenectomy	1	0	1
Antiphospholipid syndrome	0	0	0
History of malignancy	3	1	2
Blood type			
O	3	1	2
A	4	2	2
B	6	3	3
AB	0	0	0
Smoking	4	2	2

Abbreviations: *n*—number of patients; VA—ventriculoatrial.

**Table 3:** Concomitant diseases and received therapy at enrolment.

Concomitant Diseases	All CTEPH Patients, *n*	CTEPH Patients in 2019, *n*	CTEPH Patients in 2020, *n*
Coronary artery disease	8	4	4
Systemic arterial hypertension	6	2	4
Dyslipidemia	6	4	2
Thyroid disease	5	2	3
Atrial fibrillation	2	1	1
Chronic kidney disease	2	0	2
Chronic obstructive pulmonary disease	2	1	1
Diabetes	0	0	0
Obstructive sleep apnea	0	0	0
PAH-specific treatment			
PDE5i	13	6	7
Endothelin receptor antagonist	1	0	1
Treatment			
Beta blockers	9	5	4
ACEI	3	1	2
ARB	1	0	1
Potassium-sparing diuretics	13	6	7
Loop diuretics	9	3	6
Thiazide diuretics	0	0	0
Ivabradine	7	4	3
Statins	9	4	5
Anticoagulant			
Vitamin K antagonists	8	3	5
New oral anticoagulants	5	3	2

Abbreviations: PDE5i—phosphodiesterase type 5 inhibitors, ACEI—angiotensin convertase inhibitors, ARB—angiotensin receptor blockers, *n*—number of patients.

## Data Availability

The datasets used and/or analyzed during this study are available from the corresponding author upon reasonable request.
